# Preoperatively diagnosed gastric collision tumor with mixed adenocarcinoma and gastrointestinal stromal tumor: a case report and literature review

**DOI:** 10.1007/s12328-021-01343-4

**Published:** 2021-01-29

**Authors:** Kunihiko Matsuno, Yoshikazu Kanazawa, Daisuke Kakinuma, Nobutoshi Hagiwara, Fumihiko Ando, Yuka Masuda, Itsuo Fujita, Hiroki Arai, Tsutomu Nomura, Shunji Kato, Toshiro Yoshiyuki, Wei-Xia Peng, Hiroshi Yoshida

**Affiliations:** 1 Department of Gastrointestinal and Hepato-Biliary-Pancreatic Surgery, Nippon Medical School, 1-1-5, Sendagi, Bunkyo-ku, Tokyo, 113-8603 Japan; 2 Department of Integrated Diagnostic Pathology, Nippon Medical School, 1-1-5, Sendagi, Bunkyo-ku, Tokyo, 113-8603 Japan

**Keywords:** Gastric cancer, Gastrointestinal stromal tumor, Collision tumor

## Abstract

Reports of gastric collision tumors, comprising adenocarcinoma and gastrointestinal stromal tumor, are extremely rare. Here, we report the case of a 68-year-old male who was diagnosed with a lower-body, moderately differentiated, tubular-type adenocarcinoma and submucosal tumor and underwent an elective D2 distal gastrectomy. The tumor cells of the gastrointestinal stromal tumor were positive for H-caldesmon and CD117, weakly positive for smooth muscle actin and DOG-1, and negative for desmin, S-100 protein, CD31, and AE1/AE3. The tumor had grown into a mixed form of adenocarcinoma and gastrointestinal stromal tumor. Thus, we report the first case of a preoperatively diagnosed collision tumor in the stomach consisting of adenocarcinoma and gastrointestinal stromal tumor.

## Introduction

A gastric collision tumor is characterized by two tumors that are in contact with no inclusion and is relatively rare [1]. In general, adenocarcinoma accounts for 95% of the malignant neoplasms of the stomach; however, gastrointestinal stromal tumors (GISTs) have rarely been observed [2–5]. Adenocarcinoma and GIST emerge from different layers of the stomach; therefore, their collision tumor consisting of these types of cancer is rare [6]. Here, we report a very rare case of collision tumor of the stomach comprising both adenocarcinoma and GIST.

## Case report

**Fig. 1:**
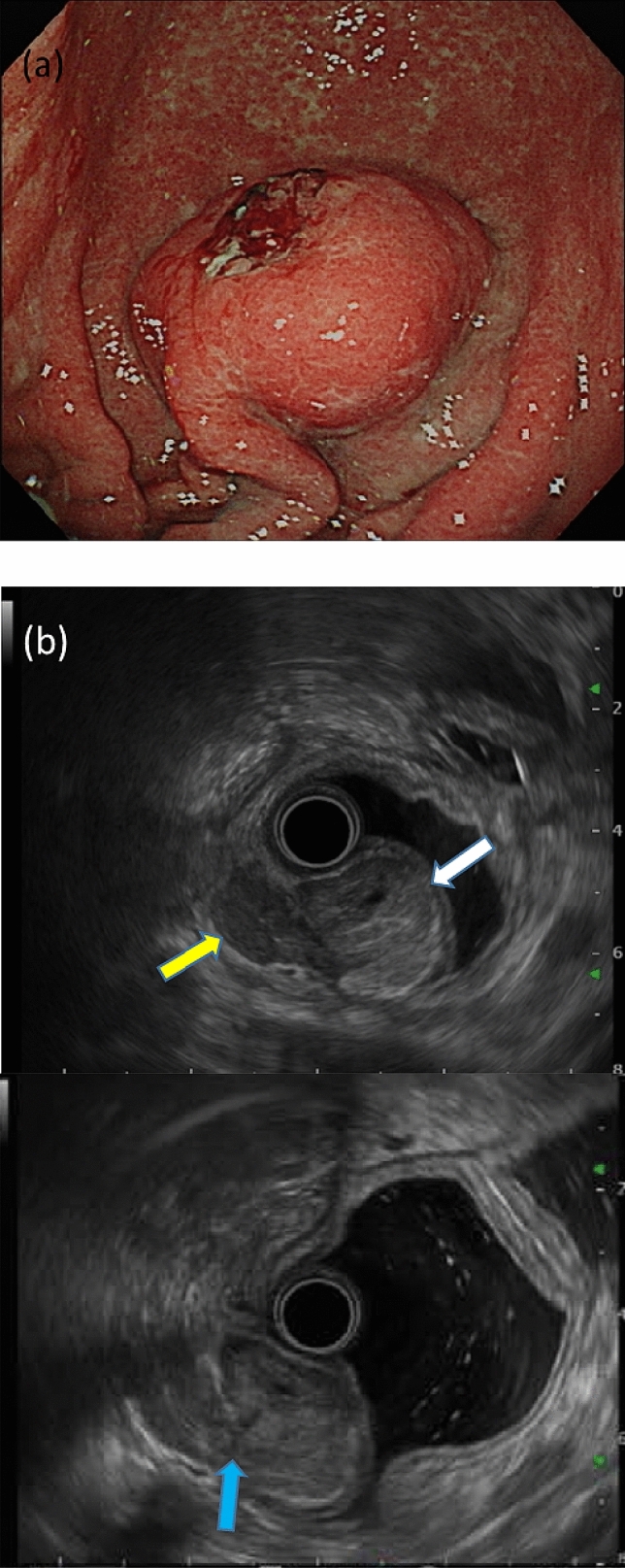
Ultrasound endoscopy suggests the possibility of collision tumor. **a** Esophagogastroduodenoscopy shows a submucosal tumor-like elevated lesion with delle. **b** Tumor with the third layer as the main locus (white arrow). Tumor with the fourth layer as the main locus (yellow arrow). The boundary obscure areas of both tumors (blue arrow)

**Fig. 2:**
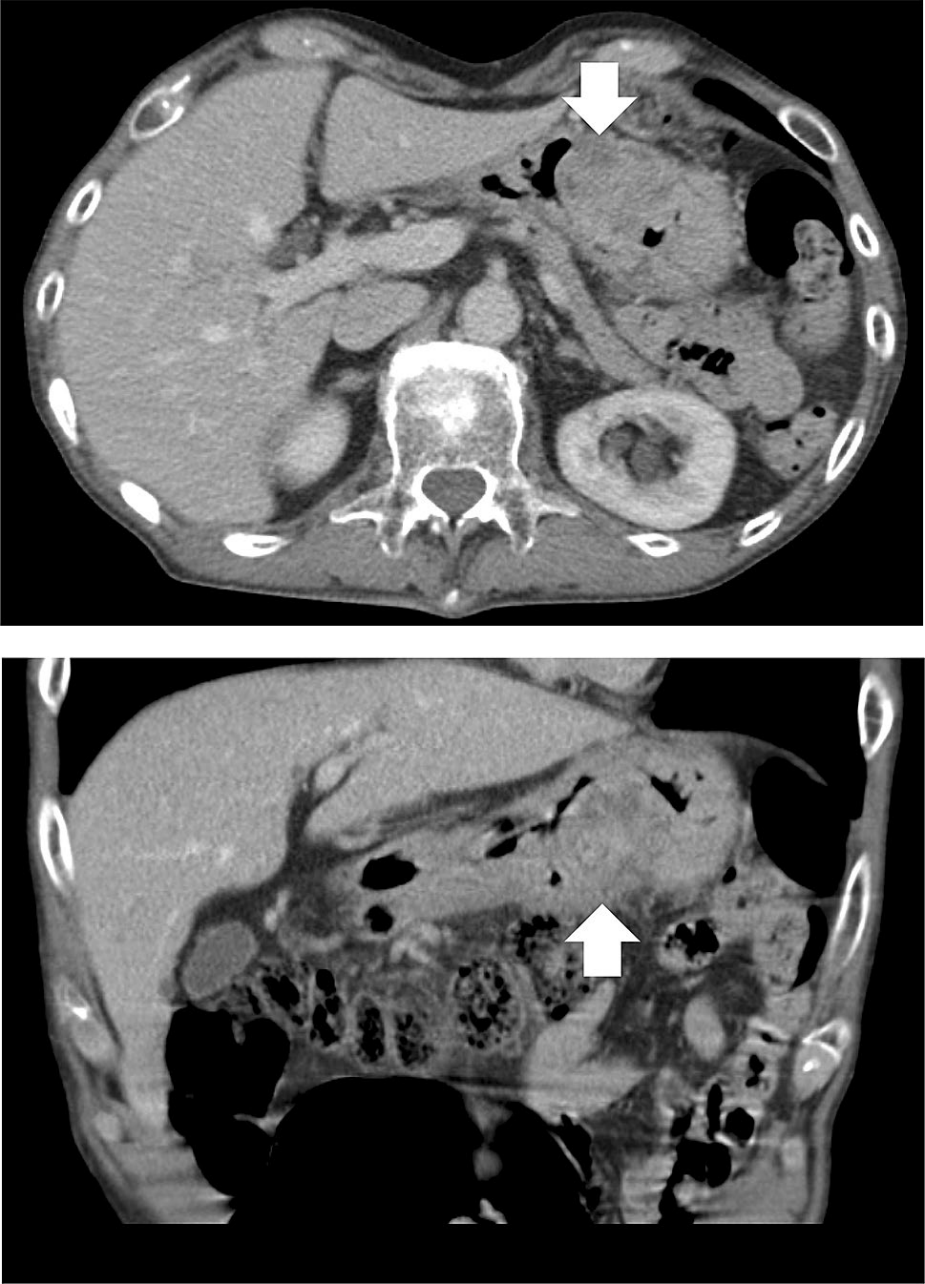
Computed tomography shows tumor with an unevenly contrasted surface of approximately 50 mm in diameter along the stomach wall (white arrows)

A 68-year-old male with a height and weight of 174.5 cm and 43.6 kg, respectively, and a primary complaint of anemia was admitted to our facility. Abdominal examination revealed that the abdominal region was leveled and soft without any pressure or pain, and no palpable masses or superficial lymph nodes were observed. Hematological tests showed a hemoglobin (Hb) level of 11.8 g/dL, indicating anemia, and no abnormalities were indicated by CEA, CA19-9, and AFP tumor markers were observed. Esophagogastroduodenoscopy revealed a submucosal tumor-like mass with delle and bridging fold in the greater curvature of the lower stomach, and the tumor was suspected as the cause of anemia (Fig. 1). Ultrasound endoscopy revealed a submucosal tumor (SMT) (with the fourth layer as the main locus) and a tumor (with the third layer as the main locus), with some areas of indistinct borders (Fig. 1b). Tissue biopsy of this tumor indicated group 5 tubular adenocarcinoma, and thoracoabdominal computed tomography (CT) showed a tumor with a mottled contrast stain in the stomach body and no regional lymph nodes or distant metastases (Fig. 2). Based on the above findings, the patient was diagnosed with collision tumor of gastric cancer and SMT (GIST was highly suspected). Gastrectomy was performed via laparotomy, and the surgical findings were a diagnosis of H0P0CY0 according to the 3rd English edition of the latest edition of the Japanese classification of gastric carcinoma issued by the Japan Gastric Cancer Society [7]. Distal gastrectomy was performed with D2 lymph node dissection according to the Japanese Gastric Cancer Treatment Guidelines [8].

**Fig. 3:**
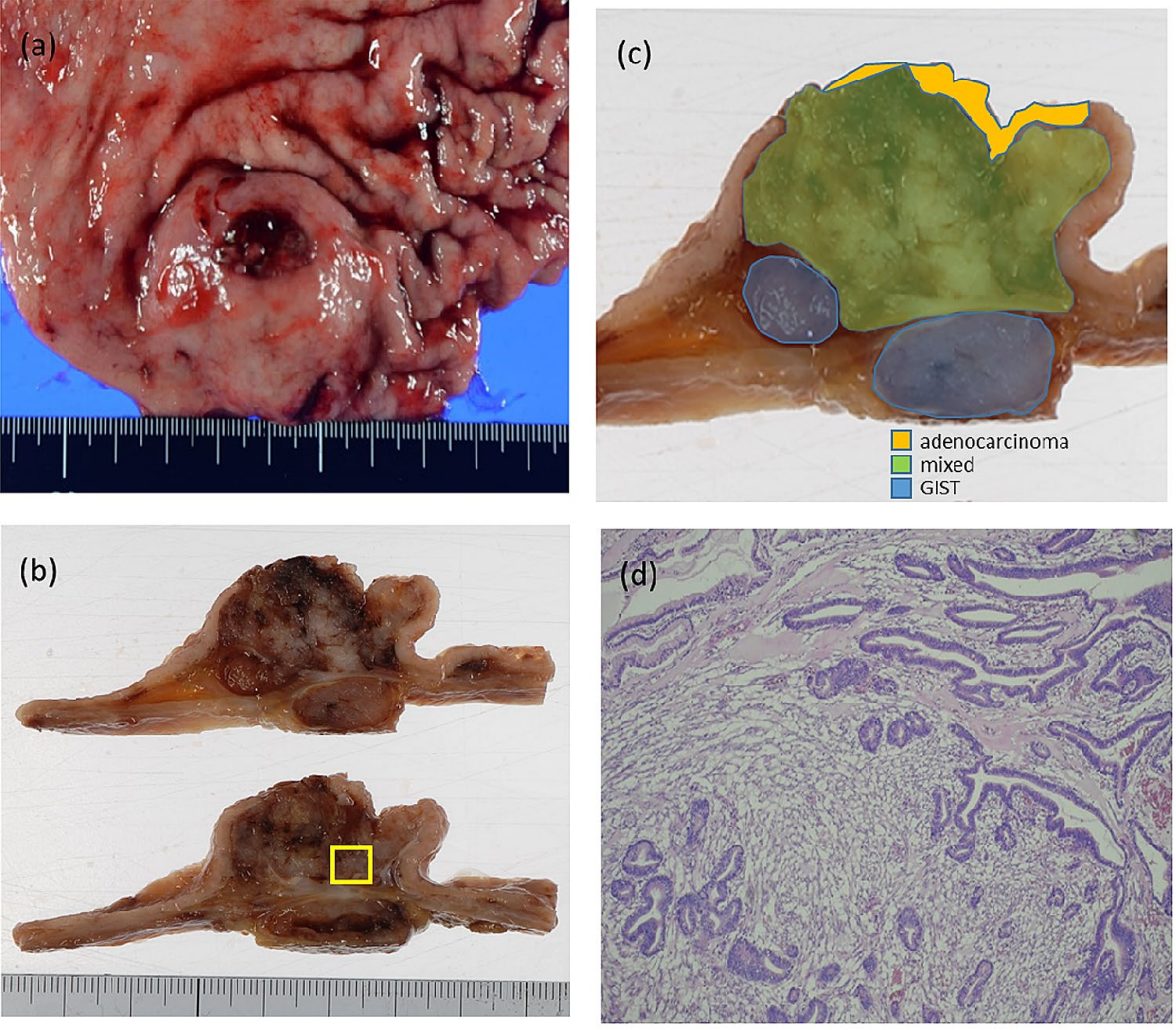
Macroscopic and microscopic findings of the resected specimens. The resected tumor shows components of both adenocarcinoma cells and spindle cells. **a** There is a submucosal tumor-like lesion with delle on the lower body of the stomach. **b** The cross-section shows an ash–white tumor with partial bleeding. **c** Schematic diagram of tumor localization. **d** Enlarged portion of the yellow square shown in **b**: pathological findings reveal that the adenocarcinoma is mixed with spindle cell tumor (hematoxylin and eosin staining)

**Fig. 4:**
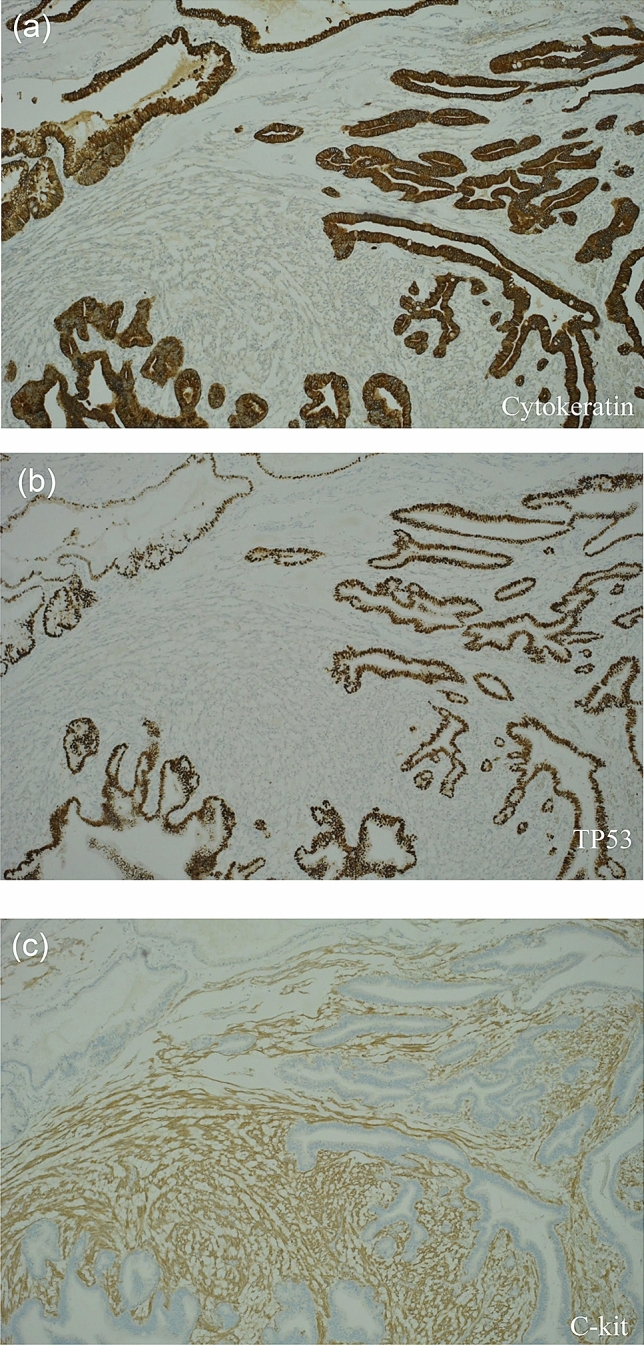
Pathological findings reveal a collision tumor comprising both adenocarcinoma and gastrointestinal stromal tumor. **a** Immunohistochemical staining demonstrates a positive reaction for cytokeratin (AE1/AE3) in the adenocarcinoma cells. **b** The nuclei of adenocarcinoma cells are positive for TP53. **c** Immunohistochemically, the spindle cell tumor is positive for C-kit

The tumor was located in the greater curvature of the antrum of the stomach and measured 50 × 45 mm in size (Fig. 3a). On the cut surface, a nodular tumor was mainly observed in the submucosal layer, and the mucosal surface was ulcerated (Fig. 3b). A large part of the tumor was observed to be mixed and some of the cancer parts and GIST alone sites were observed. (Fig. 3c). The nodular tumor was characterized by the intermingled proliferation of carcinoma cells and spindle cells (Fig. 3d). The spindle cells formed a nodular tumor in the submucosal layer and the subserosal layer. Carcinoma cells invaded the nodular tumor of the spindle cells. Pathological examination revealed Ki67 positive cells comprised 2% of the GIST. The average mitotic count was ≤ 5 mitotic figures per 50 high power fields. GIST was classified as very low risk according to the guidelines for GIST risk stratification [3]. Tubular adenocarcinoma had reached the subserosa, and no lymph node metastases were observed. The pathological findings were a diagnosis of pT3N0M0 pStageIIA, according to the 3rd English edition of the latest edition of the Japanese classification of gastric carcinoma issued by the Japan Gastric Cancer Society [7]. Immunohistochemical staining demonstrated a positive reaction for cytokeratin (AE1/AE3) in the carcinoma cells (Fig. 4a). Further, the nuclei of adenocarcinoma cells were positive for TP53 (Fig. 4b). Although the spindle cells were negative for cytokeratin (AE1/AE3) and TP53, these cells were positive for c-kit (Fig. 4c), weakly positive for DOG-1, and negative for desmin and S-100. As a result, the tumor was diagnosed as a collision tumor of GIST and adenocarcinoma. Lymph node metastasis of GIST or adenocarcinoma was not observed, and the resected margins were negative for neoplastic cells. Histologically, the intratumoral GIST sections contained different KIT exon 17 mutations, and KIT exon 17 mutation corresponding to amino acid substitution Asp820Lys was observed. On the other hand, the adenocarcinoma section detected no KIT mutation.

No complications were observed postoperatively, and the patient was discharged from the hospital on postoperative day 14. No adjuvant therapy was required for GIST, as per the international guidelines for GIST risk stratification [3]. The patient was clinically and radiographically disease-free at 2.5 years after surgery.

## Discussion

A collision tumor of adenocarcinoma and GIST in the stomach is extremely rare in gastric surgery. Only nine cases of GISTs have been reported in cancerous gastric collision tumors, including autologous cases, and these are shown in Table 1. In this report, a collision tumor of adenocarcinoma and GIST could be classified into two types: contact type wherein the tumor components are in contact with each other, and the mixed type wherein the tumor components are intermingled. We report the first case of a preoperatively diagnosed collision tumor in the stomach consisting of adenocarcinoma and GIST.

**Table 1:** Cases of collision tumor comprising gastrointestinal stromal tumor and gastric adenocarcinoma

Author	Year of publication	Sex	Age	GIST size (mm)	Histological type	Early/advance	GIST (hpf)	Collision type
Liu et al. [9]	2002	Male	70	80 × 50	Differentiated	Advance	0/50	Mixed
Katsoulis et al. [10]	2007	Female	78	100 × 80	Undifferentiated	Advance	N/A	Contact
Idema et al. [11]	2008	Male	71	50 × 40	Undifferentiated	Advance	< 5/50	Contact
Trabelsi et al. [12]	2008	Male	54	NA	Undifferentiated	Advance	0/50	Contact
Bi et al. [13]	2008	Female	73	40 × 30	Differentiated	Advance	5/50	Mixed
Toyoda et al. [14]	2009	Female	83	90	Undifferentiated	Advance	6/50	Contact
Kleist et al. [15]	2010	Female	86	60	Differentiated	Advance	< 5/50	Mixed
		Male	78	55 × 60	Undifferentiated	Advance	< 5/50	Contact
Present case	2020	Male	68	50 × 45	Differentiated	Advance	< 5/50	Mixed

*GIST* gastrointestinal stromal tumor, *N/A* not available

Meyer defined collisional tumors in 1919 [16], as two types of unrelated tumors in the same organ that come in contact or partially infiltrate each other. In 1980, Spagnolo et al. [17] established the diagnostic criteria for collisions as follows: (1) the distribution of two distinct tissue types can be clearly distinguished, (2) each tissue type can be identified at adjacent sites, and (3) both components are mixed at the collision site, implying that the portion that seems like a transition of both components could be mixed on the inside. Eight cases of collision between GIST and gastric adenocarcinoma have been reported (Table 1): five of these cases were males and three were females. The mean age of the patients was 72.3 years (range 54–86 years). In all these cases, GISTs were of low malignant potential, whereas gastric adenocarcinomas were usually advanced. We redefined Meyer's classification for collision tumors as contact type and Spagnolo's classification as a mixed type. Therefore, the classification of the cases, according to our literature review, suggested four cases of contact type and four cases, including our case, of mixed type. Regarding the diagnosis, except for this case, all reported cases were diagnosed with collision tumors after surgery. No case underwent preoperative ultrasound endoscopy, except for the present case. Pre-operative ultrasound endoscopes and ultrasound endoscopic needle biopsy are considered useful for the diagnosis of collision tumors.

Kleist et al. [15] have described a rare case of a gastric adenocarcinoma inside a GIST. They reported that this might have resulted from the dysplastic epithelium trapped inside a GIST sustaining the tumorigenic effect of the in-tumor microenvironment or tumor-to-tumor metastasis from an independent gastric adenocarcinoma. Regarding GIST and gastric cancer found in the same specimen of the surgically resected esophagogastric junction and stomach, Abraham [18] reported that in a detailed review of 150 surgical specimens that were operated for esophagogastric junction cancer, incidental GIST (median tumor diameter 1.3 mm) was identified in 10% of the specimens, with no continuity between any of them and the cancer. Kawanowa et al. [19] reported a review of 100 surgical specimens of gastric carcinoma that were operated and found GIST as small as 5 mm in 50 lesions (35 patients), 90% of which were in the gastric upper body. Although the true prevalence of these reports is unknown because the tests were performed postoperatively, micro GISTs that are not clinically problematic may be considered relatively frequent. Several cases describing various other combinations of tumors have been reported [6, 15]. Various hypotheses have been proposed about the synchronous occurrence of GIST and adenocarcinoma. Yan [20] reported that KIT and PDGFRA mutation analysis of gastric tumors, background gastric mucosa, and GIST in 15 cases, wherein gastric GIST was accidentally found in surgical specimens operated for gastric cancer, showed no genetic association. Therefore, we cannot exclude the possibility of the involvement of other unknown genes; however, if a specific factor triggered the development of both tumors, there would likely be more reports.

Here, we report the ninth case of a collision tumor of gastric adenocarcinoma and GIST. The cause of collision tumors comprising gastric adenocarcinoma and GIST has not been identified. Besides the likelihood of accidental tumors, the possibility of an identical genetic abnormality or one tumor inducing the other has been cited. Molecular genetic finding were performed in three of the eight cases in the previous article, but the only genes searched were C-kit and PDGFRA. In our case, the GIST section showed mutations in the C-kit gene; however, similar to previous reports, no mutations were observed in the cancer section. However, it cannot be ruled out that mutations in genes other than C-kit and PDGFRA could be the cause. If a search for mutations in all genes can be performed, there may be a common genetic mutation in unknown gastric cancer and GIST.

Clinically, they are usually indistinguishable from the dominant tumor type, and diagnosis is almost always determined postoperatively on histological findings. In summary, in this case, we could diagnose gastric adenocarcinoma and GIST collision tumors preoperatively using ultrasound endoscopy. Endoscopy is considered useful for diagnosis in gastric cancer with an SMT-like lesion. Although it was a very rare collision tumor, we were able to reveal the unique features and details of tumorigenesis preoperatively. When such a rare collision tumor is encountered, it should be carefully diagnosed preoperatively and examined in detail postoperatively, including genetic analysis.
